# Optimal treatment duration of bismuth-containing quadruple therapy in *Helicobacter pylori* infection: A retrospective study

**DOI:** 10.1097/MD.0000000000036310

**Published:** 2023-12-01

**Authors:** Ji Yoon Kim, Sang Gyun Kim, Soo-Jeong Cho

**Affiliations:** a Department of Internal Medicine, Liver Research Institute, Seoul National University Hospital, Seoul National University College of Medicine, Seoul, Republic of Korea.

**Keywords:** bismuth-containing quadruple therapy, eradication rate, *Helicobacter pylori*, optimal duration, treatment efficacy

## Abstract

The use of bismuth-containing quadruple therapy (BQT) in *Helicobacter pylori* eradication has been increasing. Although the recommended treatment length for BQT is 14 days, longer durations may be associated with higher rates of adverse events. The aim of this study was to evaluate the optimal duration of BQT by comparing eradication rates and adverse events among 7, 10, and 14-day regimens. A total of 328 patients treated with BQT at Seoul National University Hospital from January 2010 to May 2022 were retrospectively evaluated. The eradication rates of different treatment groups were compared using intention-to-treat (ITT) and per-protocol (PP) analyses. Baseline characteristics of the enrolled patients and adverse events were also analyzed. A total of 74, 177, and 77 patients were included in the 7-, 10-, and 14-day groups, respectively. Forty-one patients were lost during the follow-up. The eradication rates were 71.6%, 84.2%, and 80.5% (*P* = .106) by ITT, and 84.1%, 94.9%, and 92.5% (*P* = .028) by PP analysis in the 7-, 10-, and 14-day groups, respectively. The 10-day regimen showed significantly higher eradication rates than the 7-day regimen in both ITT (*P* = .024) and PP (*P* = .018) analyses. However, there were no significant differences in eradication rates between the 10- and 14-day groups in either ITT (*P* = .667) or PP (*P* = .537) analysis. Adverse event incidence was comparable among the groups (*P* = .835). Treatment with BQT for 10 days was as effective as 14 days without increasing the adverse events.

## 1. Introduction

A large proportion of the world’s population is infected with *Helicobacter pylori*, which plays a major role in the development of peptic ulcer disease, early gastric cancer (EGC), and low-grade mucosa-associated lymphoid tissue lymphoma (MALToma) of the stomach.^[1]^ *H pylori* is the most common cause of peptic ulcer disease, and eradication can lower its recurrence.^[2]^ Thus, *H pylori* eradication is highly recommended for all patients with peptic ulcer, gastric MALToma, and after endoscopic resection of EGC. *H pylori* eradication can also be considered for those with unexplained iron deficiency anemia and other associated conditions, such as gastric adenoma, functional dyspepsia, chronic atrophic gastritis, and intestinal metaplasia, but with lower levels of evidence.^[3]^

According to the 2013 revised guidelines for the diagnosis and treatment of *H pylori* infections in Korea, a proton pump inhibitor (PPI)-clarithromycin-amoxicillin regimen is recommended as the first-line therapy. When the first-line treatment fails or there is suspected clarithromycin resistance, bismuth-containing quadruple therapy (BQT) can be considered. BQT consists of a PPI, metronidazole, bismuth, and tetracycline, and is typically administered for 7 to 14 days.^[4]^ The Maastricht VI/Florence consensus, newly published in 2022, advises clinicians to prescribe BQT for 14 days unless 10 days of equally effective therapy is available.^[5]^ In support of this guideline, a meta-analysis comparing treatment durations of 1 to 3 days, 4 days, and 7 days with 10 to 14 days demonstrated a higher eradication rate from 10 to 14 days of therapy, despite a high rate of metronidazole resistance.^[6]^ Some Korean studies comparing 1 and 2 weeks of BQT also suggested that extended therapy could lead to a higher eradication rate.^[7,8]^

While increasing the length of therapy may lead to higher eradication rates, it is important to note that the probability of developing adverse events could also rise with a longer duration of treatment. Adverse events from BQT are more common than those from clarithromycin-containing triple therapy.^[9]^ Some patients can develop intolerance to BQT. Therefore, it is necessary to determine the minimum duration that can achieve the maximum eradication rate.

We retrospectively compared the eradication rates of BQT with different treatment durations (7, 10, and 14 days) in patients who failed first-line triple therapy or presented with clarithromycin-resistant infections to determine the ideal treatment period. The eradication rate, baseline characteristics of the enrolled patients, and therapy-related adverse events were compared among the groups.

## 2. Materials and methods

### 2.1. Study population

Patients treated with BQT due to persistent *H pylori* infections after first-line therapy with clarithromycin-containing triple-regimen or clarithromycin-resistant infections at Seoul National University Hospital (SNUH) from January 1, 2010, to May 30, 2022, were evaluated. The inclusion criteria for the study population were individuals with (A) a positive rapid urease test result, (B) pathologic evidence of *H pylori* infection through modified Giemsa staining, or (C) a positive ^13^C urea breath test (UBT) result. The exclusion criteria were individuals (A) on concomitant antibiotics, (B) currently on anti-coagulants, non-steroidal anti-inflammatory drugs, or steroids, (C) with critical illness, and (D) who were pregnant or breastfeeding. *H pylori* status was considered positive if one of the following tests was positive: rapid urease test (CLOtest; Delta West, Bentley, Australia), a pathological finding from an endoscopic biopsy using the Sydney system, ^13^C UBT, or DPO-PCR test (Seeplex ClaR-H. pylori ACE Detection; Seegene Institute of Life Science, Seoul, Korea).

### 2.2. Study design

This study was conducted at SNUH in a retrospective manner. The Institutional Review Board (IRB) of SNUH approved the study protocol (IRB number: H-2205-174-1329). Patients were treated for 7, 10, or 14 days based on the clinician preference. Quadruple therapy included a PPI twice daily (omeprazole 20 mg, lansoprazole 30 mg, pantoprazole 40 mg, rabeprazole 20 mg, esomeprazole 40mg, or ilaprazole 10mg) or tegoprazan 50mg daily, bismuth 300 mg twice daily, tetracycline 500 mg 4 times daily, and metronidazole 500 mg 3 times daily. The UBT was considered the primary method to assess *H pylori* eradication after the treatment due to its high availability and cost-effectiveness. Follow-up examinations were conducted at least 4 weeks after BQT completion, and PPIs were discontinued at least 2 weeks before the UBT to minimize false-negative results.

The patients were categorized into 3 groups in accordance with the treatment duration. The eradication rates were compared between the groups. The subjects’ baseline characteristics, such as age, sex, underlying diseases, and history of gastric surgery, were reviewed. The severity of adverse reactions was assessed based on physicians’ descriptions in the electronic medical record. A reaction was considered mild when the therapy could be continued without intervention, and moderate when the treatment had to be interrupted with or without additional treatment. Reactions that resulted in permanent disability or required hospital admission for potentially life-threatening events were classified as severe. Most of the information for the analyses, including the follow-up *H pylori* test results, the patient’s underlying characteristics, and adverse events, were obtained from the electronic medical record.

### 2.3. Statistical analysis

The primary outcome was the eradication status of *H pylori* identified by UBT. The *H pylori* eradication rates for each treatment group were evaluated using both intention-to-treat (ITT) and per-protocol (PP) analyses. ITT analysis included all participants, regardless of whether they received the treatment or not, while PP analysis only included participants who took the medication and followed the treatment protocol. For this study, all patients who received BQT were included in the ITT analysis, even those lost during the follow-up. The PP analysis only included participants who completed the follow-up examination after eradication therapy. The Chi-square test or Fisher exact test was performed. Baseline characteristics and adverse events were analyzed using the Chi-squared test, Fisher exact test, or analysis of variance according to the variable characteristics. A *P* value of <.05 was considered statistically significant. All statistical tests were performed using R studio 4.2.1 (https://posit.co/download/rstudio-desktop/).

## 3. Results

### 3.1. Study population

A total of 328 patients treated with BQT were enrolled in this study. There were 74, 177, and 77 patients prescribed with 7, 10, and 14 days of BQT regimen, respectively. Forty-one patients were lost during the follow-up, and their eradication status could not be identified. There were no significant differences in baseline characteristics among the 3 groups except for the prevalence of EGC (Table 1). The number of patients with EGC was significantly higher in the group with the 10-day regimen than in the other groups. Endoscopic findings such as peptic ulcer disease, MALToma, gastric adenoma, hyperplastic polyp, and subepithelial tumor were not significantly different among the 3 groups.

**Table 1:** Baseline characteristics of patients receiving bismuth quadruple therapy according to treatment duration.

	7 days (n = 74)	10 days (n = 177)	14 days (n = 77)	*P* value
Sex				
Male	42 (52.8%)	81 (45.8%)	34 (44.2%)	0.214
Female	32 (43.2%)	96 (54.2%)	43 (55.8%)	
Age	57.2 ± 13.3	60.0 ± 11.5	57.7 ± 12.3	0.812
History of prior gastric surgery	2 (2.7%)	3 (1.7%)	0 (0.0%)	0.335
Peptic ulcer	20 (27.0%)	26 (14.7%)	13 (16.9%)	0.065
EGC	5 (6.8%)	25 (14.1%)	2 (2.6%)	0.011
MALToma/DLBCL	4 (5.4%)	5 (2.8%)	5 (6.5%)	0.311
Gastric adenoma	5 (6.8%)	18 (10.2%)	4 (5.2%)	0.362
Hyperplastic polyp	0 (0.0%)	7 (4.0%)	4 (5.2%)	0.143
GIST/SET	0 (0.0%)	7 (4.0%)	1 (1.3%)	0.169
*H pylori* associated gastritis	40 (54.1%)	96 (54.2%)	50 (64.9%)	0.250

DLBCL = diffuse large B cell lymphoma, EGC = early gastric cancer, GIST = gastrointestinal stromal tumor, SET = subepithelial tumor.

### 3.2. *H pylori* eradication rate

**Figure 1.**
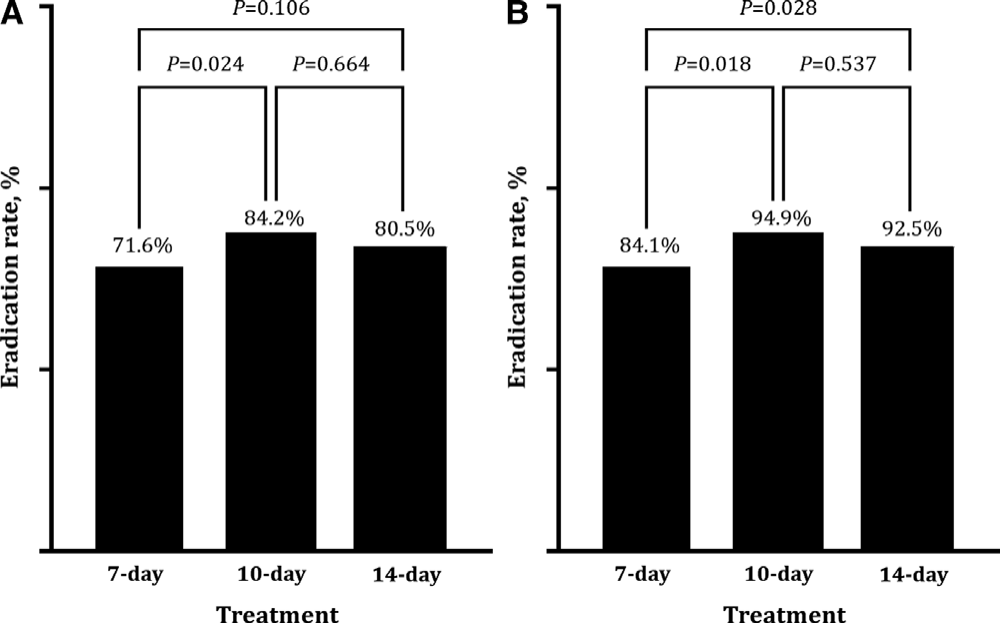
(A) ITT analyses of eradication rates according to treatment duration. (B) PP analyses of eradication rates according to treatment duration. ITT = intention-to-treat, PP = per-protocol.

In the overall comparison, the ITT eradication rate was 71.6%, 84.2%, and 80.5% for the 7-, 10-, and 14-day regimens (*P* = .106), respectively, while the PP eradication rate was 84.1%, 94.9%, and 92.5% for the 7-, 10-, and 14-day regimens (*P* = .028). In the comparison between 7-day and 10-day groups, eradication rates were significantly higher in the 10-day group by both ITT (*P* = .024) and PP (*P* = .018) analyses. In contrast, no significant difference in eradication rates was seen between the 10-day and 14-day regimen groups by ITT (*P* = .664) or PP (*P* = .537) analysis (Fig. 1A and B). Although the 10-day regimen group showed a significantly higher eradication rate than the 7-day group, there was no statistically significant difference in the eradication rate between the 10-day regimen group and the 14-day regimen group.

### 3.3. Adverse events

There were a total of 3 (4.1%), 11 (6.2%), and 3 (3.9%) adverse events observed in the 7-, 10-, and 14-day treatment groups (*P* = .460), respectively. Among these events, 2 (2.7%), 6 (3.4%), and 3 (3.9%) were classified as mild, respectively, while 1 (1.4%), 5 (2.8%), and 0 (0.0%) were classified as moderate in the 7-, 10-, and 14-day groups. There were no serious adverse events causing life-threatening reactions, permanent disability, or requiring hospital admission for immediate intervention. Most patients presented with gastrointestinal symptoms, such as nausea, vomiting, and abdominal discomfort. One patient in the 10-day regimen group experienced a moderate allergic reaction that required medical intervention (Table 2). Accordingly, there was no statistically significant difference in the incidence of adverse events among the treatment groups.

**Table 2:** Adverse events of patients receiving bismuth quadruple therapy according to treatment duration.

	7 days	10 days	14 days	*P* value
Total adverse events	3 (4.1%)	11 (6.2%)	3 (3.9%)	.460
Mild*	2 (2.7%)	6 (3.4%)	3 (3.9%)	1.000
Moderate†	1 (1.4%)	5 (2.8%)	0 (0.0%)	.357
Nausea/vomiting	1 (1.4%)	4 (2.3%)	1 (1.3%)	1.000
Epigastric pain	1 (1.4%)	6 (3.4%)	1 (1.3%)	.707
Dyspepsia	0 (0.0%)	0 (0.0%)	0 (0.0%)	
Dizziness	1 (1.4%)	0 (0.0%)	0 (0.0%)	.226
Diarrhea	0 (0.0%)	0 (0.0%)	1 (1.3%)	.460
Neuropathy	0 (0.0%)	0 (0.0%)	0 (0.0%)	
Regurgitation	0 (0.0%)	0 (0.0%)	0 (0.0%)	
Anorexia	0 (0.0%)	0 (0.0%)	0 (0.0%)	
Allergic reaction	0 (0.0%)	1 (0.6%)	0 (0.0%)	1.000

* Drug can be continued without any treatment.

† Drug was stopped and/or required treatment.

## 4. Discussion

The present study aimed to evaluate the efficacy of BQT in eradicating *H pylori* by comparing the eradication rates of different treatment durations. The results revealed that the 10-day regimen was more effective than the 7-day regimen, with *H pylori* eradication efficacy comparable to that of 14-day regimen. The baseline characteristics were similar among the 3 groups except for the prevalence of EGC, which was significantly higher in the 10-day regimen group. There was no statistically significant difference in the occurrence of adverse events among the 3 groups.

According to the 1997 Asia Pacific Consensus Conference on the management of *H pylori* infection, the ideal eradication rate should be 90% or greater by PP and 80% or greater by ITT analysis.^[10]^ Yet, the eradication rate of 7-day first-line triple-therapy from 2003 to 2012 has declined over 10 years to <80% in South Korea.^[11]^ This unsatisfactory result may be due to an increase in antibiotic resistance rates. A study analyzing the prevalence of *H pylori* antimicrobial resistance in Korea from 2003 to 2012 found a significant increase in resistance to clarithromycin, metronidazole, and levofloxacin.^[12]^ Clarithromycin resistance, which is a major cause of eradication failure, has recently increased to 17.8% in Korea based on bacterial cultures.^[13]^ Although first-line triple therapy is recommended for 7 to 14 days, there is no definite agreement on the optimal length of therapy. A study conducted in 2007 at 13 secondary to tertiary hospitals in Korea compared 7-day and 14-day triple-therapy regimens and failed to prove a higher eradication rate with a longer duration, suggesting that prolonged treatment does not necessarily improve therapeutic efficacy.^[14]^ Antibiotic resistance may play an important role in the outcome, as a longer duration of therapy does not necessarily help overcome clarithromycin resistance.

BQT is recommended for regions where clarithromycin resistance is prevalent. As in the first-line therapy, there was no universal agreement on the appropriate treatment duration for BQT prior to the Maastricht VI/Florence consensus guidelines. However, new Maastricht guidelines recommend BQT as the first-line treatment in areas with high dual clarithromycin and metronidazole resistance and prefers a 14-day regimen over a 7-day (or less) regimen.^[5]^ Not only did the prolonged regimen show a higher eradication rate, but it also demonstrated effectiveness in areas where metronidazole resistance was highly prevalent. The addition of PPI and extending the duration were both found to improve *H pylori* eradication in metronidazole-resistant strains.^[15]^ Likewise, in our study, the 10-day regimen resulted in a higher eradication rate than the 7-day regimen. The improvement in *H pylori* eradication by longer BQT suggests the possibility that extended therapy duration may be effective in overcoming metronidazole resistance. However, there was no significant difference in treatment efficacy between 14-day and 10-day regimens. This result implies that extending therapy beyond 10 days might not be effective in treating metronidazole-resistant strains unresponsive to a 10-day regimen.

Although prolonging treatment length might help overcome metronidazole resistance, it is also important to minimize the development of therapy-related adverse events. The number of adverse events reported in our study was low. Only minor to moderate adverse events were observed. However, it is widely acknowledged that adverse events associated with BQT are highly prevalent. In fact, the incidence of adverse events with BQT typically exceeds 50%.^[15]^ A recent meta-analysis of randomized trials further supports this notion, revealing significantly higher rates of adverse events with BQT compared to other eradication therapies (relative risk = 1.64; 95% confidence interval, 1.11–2.44).^[16]^ It is important to lower the occurrence of these unwanted reactions to improve treatment compliance, which could lead to higher treatment efficacy. Different studies have reported heterogeneous results on the incidence of adverse events in each treatment duration. A prospective study conducted at Seoul National University Bundang Hospital in 2010 revealed a higher rate of adverse events in the 2-week quadruple-therapy group compared to the 1-week group. This finding could be attributed to the possibility of adverse events occurring in a dose- and time-dependent manner. The study further demonstrated that major adverse events primarily manifested during the early stage of treatment, whereas minor events persisted throughout the therapy.^[7]^ A meta-analysis conducted in 2021 also demonstrated that the pooled adverse event rate of a 14-day BQT regimen was higher than that of a 10-day regimen.^[16]^ From these studies, it can be inferred that a longer treatment duration may lead to a higher incidence of unwanted treatment effects. Taken together, the findings indicate that an appropriate treatment duration of BQT is 10 days in terms of *H pylori* eradication and the incidence of associated adverse events.

This study considered antibiotic resistance as a primary cause of treatment failure, but it is important to acknowledge that other factors, such as compliance, previous BQT history, age, and sex, can also influence the success of *H pylori* eradication. With the information available on the compliance level of the patients, it was observed that good compliance was mostly associated with successful eradication, whereas poor compliance was associated with a higher rate of eradication failure (Table S1, Supplemental Digital Content 1, http://links.lww.com/MD/K824). Previous BQT treatment could also impact eradication, as 3 out of 23 treatment failure patients had undergone prior unsuccessful BQT treatment. Lastly, age and sex may also play roles as contributing factors. However, when participants were categorized into 3 age groups (≤49 years, 50–69 years, ≥70 years), no statistically significant difference was found (*P* = .109). Nevertheless, the older age group tended to exhibit a higher rate of follow-up loss and a lower rate of treatment success compared to the other 2 groups. Additionally, no statistically significant difference was observed between male and female patients (*P* = .861) (Table S2, Supplemental Digital Content 2, http://links.lww.com/MD/K825).

This study had several limitations. First, although the treatment duration was chosen according to the physician’s preference, there could be selection bias. Analysis of the baseline characteristics of the patients showed a higher proportion of EGC in the 10-day regimen group. It is possible that patients with EGC had better treatment compliance compared to those with benign diseases, which may have ultimately led to improved treatment responses. Second, because this study was conducted retrospectively, not all information was available. For instance, the development of adverse events in patients could be missed in a retrospective study. Due to the fact that adverse events were reported in up to 40% of the quadruple-therapy treatment group, it is possible that a significant proportion of adverse events may have been unidentified. More detailed analysis of the types and frequencies of adverse events associated with the BQT regimen could provide valuable information for clinicians in determining the optimal treatment regimen. Thirdly, the rapid urease test was used as the main diagnostic method for *H pylori* detection. This test is preferred due to its high convenience and credibility, as demonstrated by its reported high sensitivity and specificity, ranging from 85% to 97% and 89% to 100%, respectively.^[4]^ A study conducted in SNUH Bundang Hospital to evaluate the validity of the biopsy-based tests in detecting *H pylori* showed a sensitivity of 80.4% and a specificity of 96.7% with an accuracy of 84.2%.^[17]^ Yet, false-positive and false-negative results are possible and can be influenced by factors such as the concurrent use of antibiotics and PPIs. Although PPIs were usually prescribed after the endoscopic examination, the possibility of false-negative results cannot be completely ruled out. Thus, it is crucial to acknowledge that false test results can still occur during the analysis and should be taken into consideration during the diagnostic process. Lastly, the use of different PPIs in *H pylori* treatment can potentially affect the eradication rate of the infection. Variations in CYP2C19 and differences in intragastric pH contribute to the potential impact of different PPIs on eradication rates.^[18,19]^ Investigating the effect of specific PPIs on eradication rates provides valuable insight into optimal usage. Despite limitations in sample size and the unmatched duration of PPI use, subgroup analysis was conducted to explore potential differences among the PPIs. Neither the ITT analysis (*P* = .184) nor the PP analysis (*P* = .273) revealed statistically significant variations in eradication rates between the medications. Even after excluding the ilaprazole (n = 1) and tegoprazan (n = 3) groups due to their small sample size, ITT analysis (*P* = .190) and PP analysis (*P* = .225) still showed no significant differences (Table 3). These findings are consistent with previous studies that compared different PPIs in *H pylori* eradication, demonstrating similar efficacy across various PPIs.^[20,21]^ While the efficacy of tegoprazan in *H pylori* eradication is still being established, recent data from a phase III trial suggests effectiveness comparable to PPI-based triple-therapy.^[22]^ Despite these limitations, this study included a large number of patients who received BQT to compare the outcomes of different treatment durations, thereby strengthening the statistical power and improving the generalizability of the results. To the best of our knowledge, this was the largest study comparing 7-, 10-, and 14-day BQT regimens simultaneously, allowing for a more complete assessment of the various treatment durations.

**Table 3:** Eradication analysis based on PPIs.

ITT analysis	OM (n = 176)	LA (n = 49)	PA (n = 49)	RA (n = 23)	ES (n = 27)	TE (n = 3)	IL (n = 1)	*P* value
Follow-up loss	17 (9.7%)	8 (16.3%)	8 (16.3%)	4 (17.4%)	3 (11.1%)	1 (33.3%)	0 (0.0%)	.184
Success	149 (84.7%)	38 (77.6%)	35 (71.4%)	16 (69.6%)	23 (85.2%)	2 (66.7%)	1 (100.0%)	
Fail	10 (5.7%)	3 (6.1%)	6 (12.2%)	3 (13.0%)	1 (3.7%)	0 (0/0%)	0 (0.0%)	
TE, IL excluded								.190
PP analysis	OM (n = 159)	LA (n = 41)	PA (n = 41)	RA (n = 19)	ES (n = 24)	TE (n = 2)	IL (n = 1)	*P* value
Success	149 (93.7%)	38 (92.7%)	35 (85.4%)	16 (84.2%)	23 (95.8%)	2 (100.0%)	1 (100.0%)	.273
Fail	10 (6.3%)	3 (7.3%)	6 (14.6%)	3 (15.8%)	1 (4.2%)	0 (0.0%)	0 (0.0%)	
TE, IL excluded								.225

ES = esomeprazole, IL = ilaprazole, LA = lansoprazole, OM = omeprazole, PA = pantoprazole, PPI = proton pump inhibitor, RA = rabeprazole, TE = tegoprazan.

## 5. Conclusions

In this study, we observed that the 10-day regimen demonstrated a higher eradication rate compared to the 7-day regimen, and there was no statistically significant difference between the 10-day regimen and the 14-day regimen in terms of *H pylori* eradication rate. These findings suggest that prolonging the treatment duration may help overcome metronidazole resistance and improve the overall eradication rate. The results of our study suggest that a 10-day course of BQT may be considered the optimal duration for *H pylori* eradication as it demonstrated the highest efficacy in eradicating the infection while minimizing the occurrence of adverse events.

## Author contributions

**Conceptualization:** Soo-Jeong Cho, Sang Gyun Kim.

**Data curation:** Ji Yoon Kim.

**Formal analysis:** Ji Yoon Kim, Soo-Jeong Cho

**Funding acquisition:** Soo-Jeong Cho.

**Methodology:** Soo-Jeong Cho.

**Project administration:** Soo-Jeong Cho.

**Supervision:** Soo-Jeong Cho, Sang Gyun Kim.

**Validation:** Ji Yoon Kim

**Visualization:** Ji Yoon Kim

**Writing – original draft:** Ji Yoon Kim.

**Writing – review & editing:** Ji Yoon Kim, Soo-Jeong Cho.

## Supplementary Material




