# Supplementary material for: Optimal treatment duration of bismuth-containing quadruple therapy in Helicobacter pylori infection: A retrospective study
Source: Medicine (Baltimore). 2023 Dec 1;102(48):e36310. doi: 10.1097/MD.0000000000036310 (PMC10695568; doi:10.1097/MD.0000000000036310)
Supplement: Supplementary file 1 [file medi-102-e36310-s001.docx]

**Supplementary Table 1.** Compliance

|  | Good  (n=278) | Poor  (n=9) |
| --- | --- | --- |
| Successful eradication | 261/278 (93.9%) | 3/9 (33.3%) |
| Failed eradication | 17/278 (6.1%) | 6/9 (66.7%) |
