# Supplementary material for: Optimal treatment duration of bismuth-containing quadruple therapy in Helicobacter pylori infection: A retrospective study
Source: Medicine (Baltimore). 2023 Dec 1;102(48):e36310. doi: 10.1097/MD.0000000000036310 (PMC10695568; doi:10.1097/MD.0000000000036310)
Supplement: Supplementary file 2 [file medi-102-e36310-s002.docx]

**Supplementary Table 2.** Eradication analysis based on age and sex

| **Age** | | | | | | |
| --- | --- | --- | --- | --- | --- | --- |
|  | **≤49 years**  **(n=71)** | **50-60 years**  **(n=197)** | | **≥70 years**  **(n=60)** | | **p-value** |
| **Follow-up loss** | 10/71 (14.1%) | 18/197 (9.1%) | | 13/60 (21.7%) | | 0.109 |
| **Success** | 58/71 (81.7%) | 163/197 (82.7%) | | 43/60 (71.7%) | |  |
| **Fail** | 3/71 (4.2%) | 16/197 (8.1%) | | 4/60 (6.7%) | |  |
|  | | | | | | |
| **Sex** | | | | | | |
|  | **Female**  **(n=157)** | | **Male**  **(n=171)** | | **p-value** | |
| **Follow-up loss** | 18/157 (11.5%) | | 23/171 (13.5%) | | 0.861 | |
| **Success** | 128/157 (81.5%) | | 136/171 (79.5%) | |  | |
| **Fail** | 11/157 (7.0%) | | 12/171 (7.0%) | |  | |
